# Judgments of warmth and competence in a computerized paradigm: Little evidence of proposed impression formation asymmetries

**DOI:** 10.1371/journal.pone.0175210

**Published:** 2017-04-07

**Authors:** Øyvind Jørgensen, Martin Bäckström, Fredrik Björklund

**Affiliations:** 1 Centre for Psychology, Kristianstad University, Kristianstad, Sweden; 2 Department of Psychology, Lund University, Lund, Sweden; Institut Català de Paleoecologia Humana i Evolució Social (IPHES), SPAIN

## Abstract

Much of what we know concerning impression formation is based on experimental methods where the participant receives a list of traits or behaviors and is asked to make trait judgments or meta-cognitive judgments. The present study aimed to put some well-known effects from the impression formation literature to a test in a more dynamic computerized environment, more akin to many real world impression formation scenarios. In three studies participants were introduced to multiple target persons. They were given information about the target persons’ behavior, one at a time, while making ratings of their warmth and competence, and their probability of performing related behaviors in the future. In neither of the studies the negativity effect of warmth or the positivity effect of competence were reproduced.

## Introduction

The present research concerns some classic findings from research that has attempted to model impression formation, where it has been found that information related to coldness receives higher weight than information related to warmth, and information related to competence more than information related to incompetence [1]. We approach the asymmetry effects with newer experimental tools, with the aim to replicate them in a context were people form impressions of multiple targets at once, and were they receive one piece of information about each target at the time.

### Warmth and competence as fundamental dimensions in impression formation

When attending a social gathering, such as a party with many unfamiliar people, we receive bits and pieces of information about the other guests to gradually form an impression of them. When we form an impression of a person, the trait ascriptions we make tend to fall along two general, separate dimensions. When we meet a person for the first time, we want to know his or her intentions. Is the person trustworthy, tolerant, friendly and moral? This is the warmth dimension. We also want to know if the person is capable of fulfilling his or her intentions. Is the person intelligent, competitive and assertive? This is the competence dimension. The warmth and competence dimensions have been identified in both research on how we perceive individuals and how we perceive groups, and appear across different cultures [2–3]. Researchers have labeled these dimensions differently (e.g. communion and agency, morality and capacities) but we use the terms adopted by Fiske, Cuddy, Glick, and Xu [4], namely warmth and competence.

Research suggests that the warmth dimension generally receives higher weight than competence in trait inferences and when forming a global impression of a person [5–6]. Furthermore, when predicting future behavior, information along the warmth dimension seems to have a larger effect on the estimation compared to information along the competence dimension [7]. This is one of the findings that we intend to return to in the present research, to see if it can be recreated when the perceiver receives several pieces of behavioral information regarding the target person, in a sequence.

### Asymmetry effects in impression formation

In a much cited chapter, Peeters and Czapinski [8] argue that negative information generally is more impactful than positive information in person perception. This positive-negative asymmetry effect has been supported by other research [7, 9]. In addition though, it is proposed that the content of the information (e.g. warmth or competence) and valence of the information have interactive effects. It is a central idea in impression formation research that warmth-related and competence-related information have asymmetric effects on trait judgments, depending on the valence on the information. In an influential paper Reeder [10] suggests that since both cold (immoral) and warm persons are expected to perform warm behaviors at times, cold behavior is a stronger indicator that the person is indeed cold. Warm (moral) persons should refrain from performing cold behaviors, however. Accordingly, a target person should need to show fewer examples of cold behaviors in order to be perceived by observers as cold, compared to warm behaviors, which is also supported by empirical evidence [11–13]. This negativity effect is typical for situations where the information about target persons pertains to the warmth dimension [1]. There is also evidence of asymmetry in trait inferences regarding the competence dimension, but on the opposite end of the valence dimension [14]. Whereas both competent and incompetent persons will, at times, fail and show incompetence, it should be rarer for incompetent persons to demonstrate competent behaviors [10]. Therefore, a competent behavior should be more diagnostic of competence than an incompetent behavior. This positivity effect is typical for situations where the information about target persons pertains to the competence dimension [1].

Previous studies of asymmetry effects in impression formation have relied primarily on metacognitive judgments (judgments of implications of different traits), and on trait inferences from behavioral information that is presented in a list. In the present research we introduce a new methodological paradigm. Participants will receive relatively normal everyday (non-extreme) behavioral information, one behavior at a time, about several different targets and form impressions of them. They will also be asked to judge the probability that a given person performs a given behavior. This experimental setup will be a closer model of many real life situations in that participants will form an impression of several individuals in parallel, and in that all information about a target is not given at the same time.

### Hypotheses

Based on the previous work on asymmetry effects in impression formation, the following hypotheses will be tested in this study with a new paradigm:

1. Ratings of a target’s warmth will be influenced more by cold behaviors than by warm behaviors. The hypothesis is tested in experiment 1 and 3.
2. Ratings of a target’s competence will be influenced more by competent behaviors than by incompetent behaviors. The hypothesis is tested in experiment 2 and 3.

## Experiment 1 –the warmth dimension

### Method

#### Ethics statement

All studies were approved by the regional ethics board in Lund, Sweden (Regionala etikprövningsnämnden Lund). Before the start of the experiments participants filled out a written informed consent that informed them of the goals of the experiment, how the data would be stored, and that they could abandon the experiment at any time. No participants abandoned the experiments.

#### Participants

Seventy-two university students, 36 female and 36 male, were recruited at Lund University campus and volunteered to participate. They received a cinema ticket for their participation.

#### Materials and procedure

The entire study was administered in a computer application in which participants received information about eight target persons, presented with fictive names and a facial picture. The program was created based on the Windows Presentation Manager and was developed from scratch in this environment using C# code. The target persons varied in sex, age and ethnicity; four were male and four female, four were younger (mean age 23 years) and four older (mean age 55 years), and four were Caucasian and four non-Caucasian (from Africa, Asia and South America). The pictures were taken from the Productive Aging Laboratory (PAL) Face Database [15]. Participants received information about 10 behaviors that each target person had conducted, one behavior at the time. Some behaviors indicated warmth, other behaviors indicated coldness. Some behaviors were fairly warm, others fairly cold, and some behaviors neutral (neither warm nor cold). To make sure that cold and warm behaviors were clearly separated, and unrelated to competence, 167 behavioral sentences were pretested. They were rated on warmth by 10 judges, and also whether they related to competence or not. The first response was given on a graded scale (1 (very cold)– 9 (very warm), and the second response was given as yes or no (relevant for competence? Yes/no). Based on their mean ratings the behavioral sentences were categorized as cold, fairly cold, neutral, fairly warm, or warm; see Table 1 (The full stimuli can be requested from the first author). The photographs of the stimulus persons were also rated in a prestudy, by 10 other raters. There were 60 photos which were rated on 9-point scales with regard to warmth, coldness and attractiveness. The most neutral photographs were chosen for the experiment.

**Table 1:** Examples of warmth and competence-related behaviors, including cut-off scores for each category.

Category	Cut-offs	Example
Warm	> 6.4	[name] was the first to help when an old lady fell in the street
Fairly warm	5.8–6.4	[name] got someone else to take care of the dog while the guests where visiting, since he/she knew that the guests did not like the dog
Neutral	4.2–5.8	[name] went for a walk after work
Fairly cold	3.6–4.2	[name] came late to an important meeting and blamed it on the bus being late, when in fact it was not
Cold	< 3.6	[name] stopped calling his/her friend after the friend had developed a drinking problem
Competent	> 6.4	[name] always won when the friends gathered to play “Trivial Pursuit”
Fairly competent	5.8–6.4	[name] did not need a shopping list because he/she memorized all the groceries
Fairly inompetent	3.6–4.2	When [name] met the new employer for the first time, he/she suddenly got tongue-tied
Incompetent	< 3.6	[name] forgot to bring warm clothes for the winter holiday

*Note*. The cut-offs for each behavioral category are shown. One behavior from each behavioral category is included.

Each participant received information about four target persons that were mainly warm, always presented in the following order: neutral; fairly warm; neutral; fairly cold; fairly warm; warm; neutral; fairly cold; fairly warm; warm. The other four targets were mainly cold (neutral; fairly cold; neutral; fairly warm; fairly cold; cold; neutral; fairly warm; fairly cold; cold). The computer application was balanced so that each of the eight target category combinations (e.g. Stina, a young Caucasian woman) was presented as mainly warm to half of the participants, while the same target was presented as mainly cold to the other half.

Participants were instructed that the study concerned impression formation. The instructions said that “Try to imagine that you have just moved into a residential area, and you meet a lot of new people who you are trying to form an impression of. You will make judgments of persons based on information about what they have done.” The participant’s task was to rate, after each piece of information about a target´s (neighbor’s) behavior, how warm they considered the target person to be, on a scrollbar from 0–100 (0 was labelled “Very cold”, 50 “Neutral” and 100 “Very warm”). On the first trial for each target, the scrollbar was always set to 50. Participants first received one piece of information about one target, and on the next trial information about a behavior that another target had conducted. The target persons were presented in a random order, but each of the eight targets was always presented within eight trials. Participants were assisted in that the toolbar was put at the same value as their last rating of that particular target. For example, if you previously rated Stina at 60, the toolbar would be set to 60 the next time Stina was to be rated.

After receiving information about 10 behaviors for each target, after rating each target ten times, participants continued with the next task, which was to estimate how probable it was that the target persons would conduct certain warmth-related behaviors in the future. The stimuli was pretested (N = 10), and behaviors with a mean rating as clearly warm or clearly cold (and rated as not competence-related) were used in the study. Half of the behaviors were considered cold (e.g. take credit for another person’s work), the other half warm (e.g. help an ill neighbor with their laundry). There were two future warmth-related and two future coldness-related behaviors for each of the eight target persons. Probability was rated on a scrollbar from 0–100%. The target persons were presented in a random order, but each of the eight targets was always presented within eight trials. As they could use the information that they had received earlier about each target as a basis for their judgment, the warmth rating tasks bears resemblance to real life situations in which we encounter many people at once and gather bits and pieces of information about them during conversation or behavioral observation, and the probability ratings task bears resemblance to the situation where we later on try to recall the target persons’ personality.

### Results and discussion

In order to test the hypothesis that information about cold behaviors will influence warmth ratings more than warm behaviors, the gradual impression of the targets were analyzed. The first behavior for each target was always neutral (irrelevant to warmth) and was used as a baseline, and a new variable labelled *relative rating* was created, which represents the amount of change from the first impression as the participant starts to receive more and more information about the target. For example, a participant who put the scrollbar on 55 on the first rating of a target and then put the scrollbar on 60 after the next piece of information would get a relative rating score of 5 on the second trial for that target. Another variable labelled *development warmth* was created in order to account for the amount of warm or cold behavioral information the participant had received about a target at a particular time. For a warm target for instance, a neutral behavior was coded as 0, a fairly warm behavior as 1, a warm behavior as 2 and a fairly cold behavior as -1 (e.g. the behaviors fairly warm-warm-neutral would be coded 1-3-3).

#### Information signaling competence vs. incompetence

**Fig 1:**
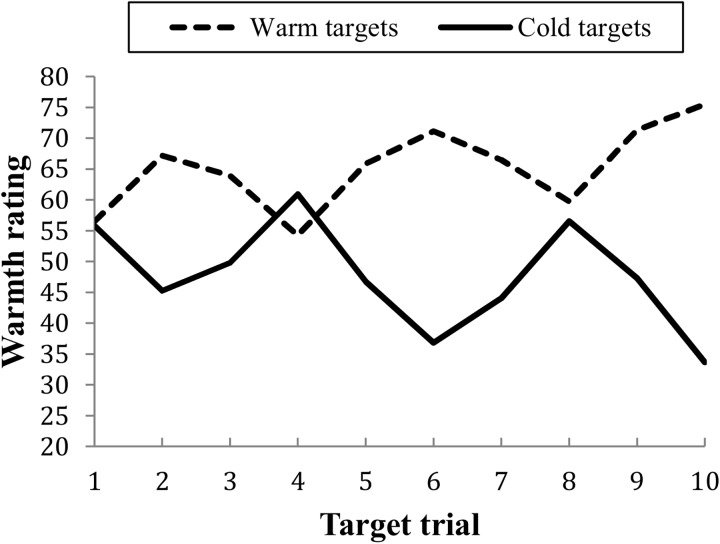
Warmth ratings across the 10 trials for mainly warm and mainly cold targets.

First the strength of the relationship between behavioral information and impressions were examined separately for the mainly warm and the mainly cold targets. The correlations between development warmth and relative ratings showed that information about cold behaviors (*r* = .36) did not influence warmth ratings more than information about warm behaviors (*r* = .37). Another way to test how much influence the warm and cold behaviors had on the impressions is to compare how much the rating of the warm and cold targets had been altered from the first ratings (only neutral information) to the last ratings of the targets. Warm targets were rated as more warm on the last rating than on the first rating; *M*_Diff_ = 18.97, *SD* = 9.46, *t*(71) = 17.01, *p* < .001, and cold targets as more cold on the last compared to the first rating; *M*_Diff_ = -22.11, *SD* = 14.39, *t*(71) = 13.03, *p* < .001 (Fig 1). The change in ratings was not significantly larger for the cold targets compared to the warm targets; *t*(71) = 1.70, *p* = .092.

#### Probability ratings

**Fig 2:**
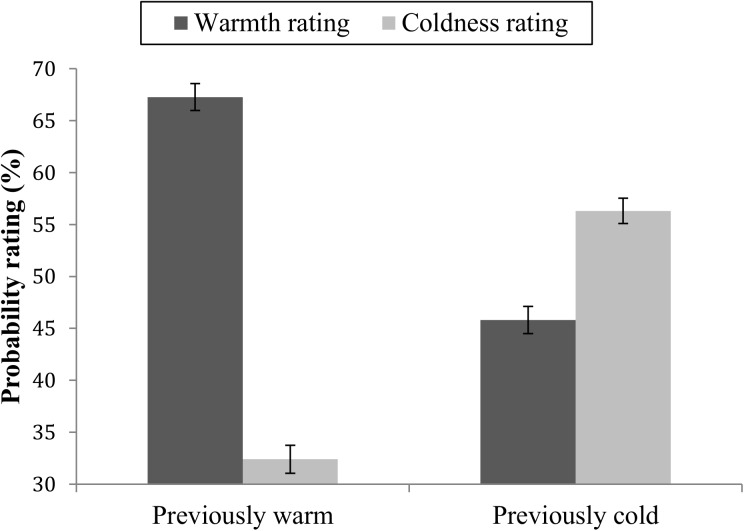
Mean probability ratings of future warm and future cold behaviors. Ratings of targets with previously mainly warm or mainly cold behavior. Error bars represent standard errors.

Probability ratings for future warm and cold behaviors were analyzed in a 2 × 2 repeated measures ANOVA with target warmth (previously warm vs. previously cold target based on past behaviors) and warmth rating (future warm vs. future cold behaviors) as within-group factors and probability ratings as the dependent variable. There was no main effect of target warmth: *F*(1,71) = 2.74, *p* = .102, η^2^ = 0.03. There was a main effect of warmth rating: *F*(1,71) = 69.36, *p* < .001, η^2^ = 0.49, where warm behaviors (*M* = 56.53, *SE* = 0.80) overall were rated as more likely than cold behaviors (*M* = 44.35, *SE* = 0.92). The target warmth × warmth rating interaction was significant: *F*(1,71) = 161.22, *p* < .001, η^2^ = 0.69 (Fig 2). In order to test whether warm or cold previous behaviors had more influence on probability ratings of future behaviors, pairwise comparisons were calculated to study the mean difference in warm vs. cold ratings between warm and cold targets. The analysis showed that warm targets were rated as much more likely to perform warm (*M* = 67.26, *SE* = 1.29) than cold (*M* = 32.39, *SE* = 1.35) behaviors, *M*_Diff_ = 34.87, *SE* = 2.40, *p* < .001. Cold targets were rated as more likely to perform cold future behaviors (*M* = 56.30, *SE* = 1.21) than warm behaviors (*M* = 45.79, *SE* = 1.31), *M*_Diff_ = -10.50, *SE* = 2.21). The mean difference in probability ratings were significantly larger for warm than cold targets: *M* = 24.36, *SD* = 24.82, (*t*(71) = 8.32, *p* < .001). This shows that information about previous warm behaviors influenced ratings of future behaviors more than information about previous cold behaviors.

Taken together, the results show that participants used the positively valenced information (warm behaviors) and the negatively valenced information (cold behaviors) to a comparable degree in the first rating task, when they gradually formed an impression of the targets as they kept receiving more information about them. When participants had to remember their targets and rate the likelihood that they would conduct future warm and cold behaviors, the results did not support the hypothesis that cold behaviors should influence impressions more. The results rather show that the warm behaviors influenced probability ratings more. This is not in line with the asymmetry model proposed by Reeder [10], where it is suggested that cold behaviors are stronger indicators of a person’s level of warmth than are warm behaviors, and cause stronger trait inferences.

In study 2, the aim was to test hypothesis 2, namely that competent behaviors will influence impressions of a person’s competence level more than incompetent behavior. The design was similar to the one used in study 1.

## Experiment 2 –the competence dimension

### Method

#### Participants

Ninety-six university students, 48 female and 48 male, were recruited at Lund University campus and volunteered to participate in the experiment. They received a cinema ticket for their participation.

#### Materials and procedure

The design of experiment 2 was identical to that of experiment 1. The same computer application was used, but instead of the warmth-dimension the competence-dimension in impression formation was studied. A pretest similar to the one described in experiment 1 was used to identify behaviors of varying degree of competence (see Table 1). Participants received information about mainly competent and mainly incompetent targets of varying age, sex and ethnicity, and were asked to rate the targets’ competence level on a scrollbar from 0–100, where 0 was labelled “Very incompetent”, 50 “Neutral” and 100 “Very competent”. Thereafter, participants were asked to rate the probability of two future competence-related (e.g. received several assignments at once, but managed to keep track of them all) and two future incompetence-related behaviors (e.g. hands in an assignment with several careless mistakes) for each of the eight targets.

### Results and discussion

As in the previous experiment, we studied how impressions developed from the initial estimate of the target, and a relative target rating was computed. Akin to study 1, a development competence and a relative rating variable was computed (see study 1 for details).

#### Information signaling warmth vs. coldness

**Fig 3:**
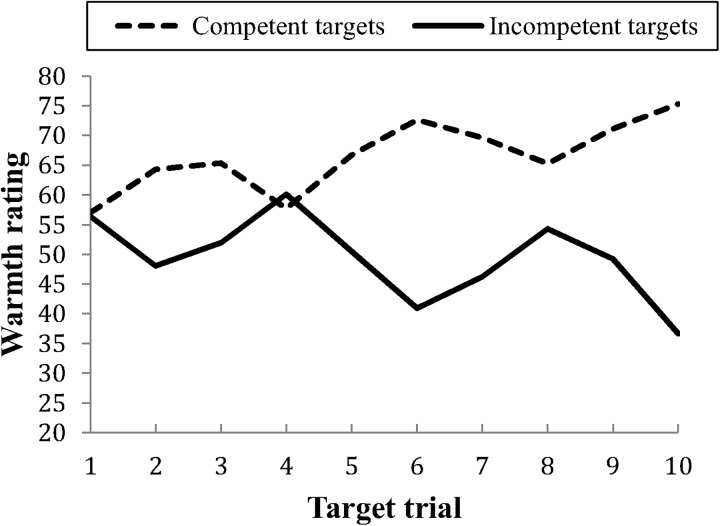
Competence ratings across the 10 trials for mainly competent and mainly incompetent targets.

Hypothesis 2 predicted that ratings of competence would be more influenced by information about competent behaviors than incompetent behaviors. Separate correlations were examined for the mainly competent and the mainly incompetent targets. The competent and incompetent target information had an equal amount of influence on change in target ratings, showing that information about competent behaviors (*r* = .36) did not influence ratings more than information about incompetent behaviors (*r* = .37). We compared how much the ratings of the competent and incompetent targets had been altered between the first rating (only neutral information) and the last rating of each target. Competent targets were rated as more competent on the last compared to the first trial; *M*_Diff_ = 18.31, *SD* = 11.16, *t*(95) = 15.66, *p* < .001. Incompetent targets were rated as more incompetent on the last compared to the first trial; *M*_Diff_ = -19.79, *SD* = 17.08, *t*(94) = 11.29, *p* < .001 (Fig 3). The absolute change in ratings was not significantly different for competent and incompetent targets; *t*(94) = 0.74, *p* = .459.

#### Probability ratings

**Fig 4:**
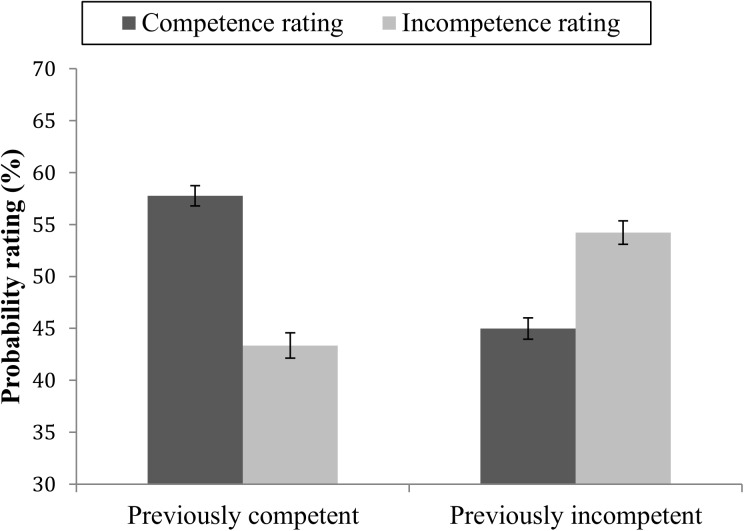
Mean probability ratings of future competent and future incompetent behaviors. Ratings of targets with previously mainly competent or mainly incompetent behavior. Error bars represent standard errors.

As in experiment 1, probability ratings were analyzed in a repeated measure ANOVA with target competence (previously competent vs. previously incompetent) and competence rating (future competent vs. future incompetent) as within-subject variables. There was no main effect of target competence (*F*(1,95) = 1.39, *p* = .240, η^2^ = 0.01), but there was a near-significant main effect of competence rating ((*F*(1,95) = 3.80, *p* = .054, η^2^ = 0.03) in that competent behaviors overall were rated as somewhat more likely to be conducted (*M* = 51.37, *SE* = 0.71) than incompetent behaviors (*M* = 48.77, *SE* = 0.93). As expected, a significant target competence × competence rating interaction was found (*F*(1,95) = 101.62, *p* < .001, η^2^ = 0.51, Fig 4). Pairwise comparisons showed that differences in probability ratings of competent vs incompetent behaviors were somewhat larger for targets who had previously shown competence (*M*_Diff_ = 14.42, *SE* = 1.81, *p* < .001) than for targets who had previously shown incompetence (*M*_Diff_ = -9.23, *SE* = 1.73, *p* < .001). The difference was near significant; *M* = 5.19, *SD* = 26.06, (*t*(95) = 1.95, *p* = .054.

Taken together, the results from study 2 do not support hypothesis 2, that competent behavior is given more weight in judgments of a person’s level of competence, compared to incompetent behavior.

## Experiment 3 –the warmth and the competence dimension together

In the two studies reported so far, participants received information about either warmth or competence, and only rated the targets on that dimension. In study 3, a more complex design was used where participants received behavioral information about both warmth and competence, and rated each target on both dimensions. This change implies that the perceivers’ context reflected the complexity of reality even more, since people’s real life behavior actually varies on both warmth and competence. The study was designed to test asymmetry effects, and we had no hypotheses regarding the effects of warmth on competence ratings or vice versa. If the asymmetry effect is not present in this context either, this would be a further indication that some boundary conditions apply for asymmetry effects in impression formation.

### Method

#### Participants

Seventy-two university students, 36 female and 36 male, were recruited at the university campus and volunteered to participate. They received a cinema ticket for their participation.

#### Materials and procedure

The design was similar to that of the previous experiments. The stimulus materials were taken from study 1 and 2. The important difference was that the information participants received about each target included behaviors along both the warmth and the competence dimension. Some targets were mainly warm and competent, some cold and incompetent, some warm and incompetent and others cold and incompetent. Each target was described by 17 different behaviors in a fixed order. If the target was mainly warm and competent, the behaviors were as follows: neutral; fairly competent; fairly warm; neutral; fairly incompetent; fairly cold; fairly competent; fairly warm; competent; warm; neutral; fairly incompetent; fairly cold; fairly competent; fairly warm; competent; warm. If the target was mainly warm and incompetent, the behaviors were presented as follows: neutral; fairly incompetent; fairly warm; neutral; fairly competent; fairly cold; fairly incompetent; fairly warm; incompetent; warm; neutral; fairly competent; fairly cold; fairly incompetent; fairly warm; incompetent; warm. A mainly cold and competent target was presented as follows: neutral; fairly competent; fairly cold; neutral; fairly incompetent; fairly warm; fairly competent; fairly cold; competent; cold; neutral; fairly incompetent; fairly warm; fairly competent; fairly cold; competent; cold. And finally, a mainly cold and incompetent target was presented as follows: neutral; fairly incompetent; fairly cold; neutral; fairly competent; fairly warm; fairly incompetent; fairly cold; incompetent; cold; neutral; fairly competent; fairly warm; fairly incompetent; fairly cold; incompetent; cold.

As in the previous experiments targets varied in gender, age and ethnicity, so that each combination of target group (male and female, young and old, Caucasian and non-Caucasian) and each of the four combinations of warmth and competence appeared in equal proportion. Thereafter, participants were asked to rate the probability of two future warmth-related, two future coldness-related, two future competence-related and two future incompetence-related behaviors for each of the eight targets.

### Results and discussion

**Fig 5:**
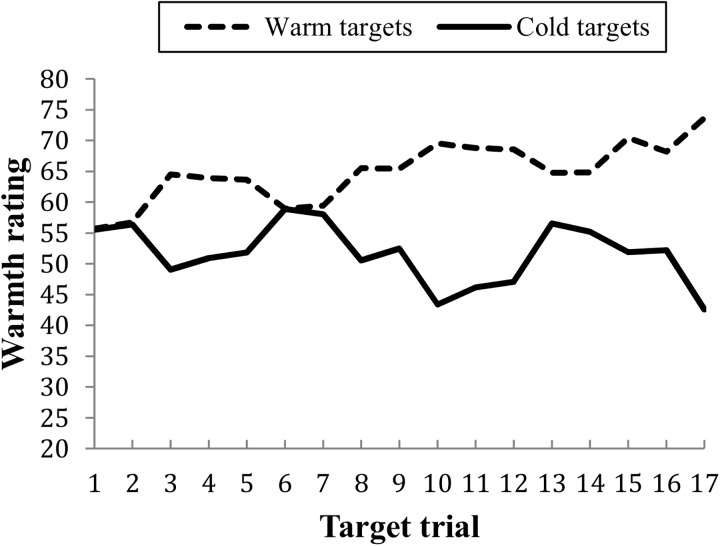
Warmth ratings across the 17 trials for mainly warm and mainly cold targets.

First, we explored how the behavioral information influenced warmth ratings. The patterns are shown in Fig 5. A standard multiple regression was performed with relative ratings as the dependent variable and developed warmth and developed competence as independent variables. As expected, behavior along the warmth dimension strongly influenced warmth ratings (Table 2). Information about competence did not influence warmth ratings. There was no developed warmth × developed competence interaction effect on warmth ratings. As expected, the warm targets were rated significantly more warm in the last trial compared to the first trial; mean change = 17.96, *SD* = 9.91, *t*(71) = 15.37, *p* < .001, and cold targets as more cold on the last than the first rating; mean change = 12.91, *SD* = 15.29, *t*(71) = 7.16, *p* < .001. The mean difference between the first and the last ratings were significantly larger for the warm than for the cold targets; *t*(71) = 3.05, *p* = .003.

**Table 2:** Standardized beta coefficients for the predictors of perceived warmth and competence.

Predictors	Warmth ratings	Competence ratings
Target warmth	0.45**	0.07**
Target competence	-0.01	0.41**
Warmth × Competence	-0.00	0.03**
*R*^2^	0.20	0.17
*F*	852.02	703.71

*Note*. Summary of results from two separate multiple regression analyses, one with warmth ratings as the dependent variable and the other with competence ratings as the dependent variable. Target warmth = How much warmth/coldness a target has shown. Target competence = How much competence/incompetence a target has shown. Warmth × Competence = The interaction effect of target warmth and target competence. Standardized coefficients are reported.

** *p* < .001

**Fig 6:**
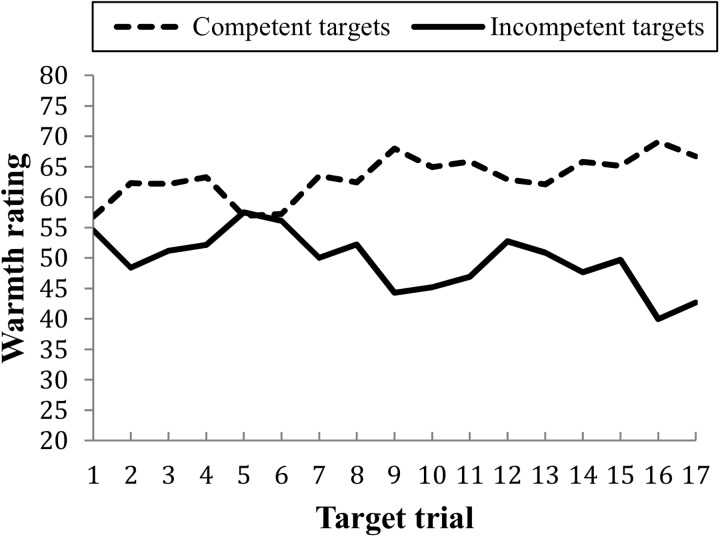
Competence ratings across the 17 trials for mainly competent and mainly incompetent targets.

Similar analyses were conducted for the competence ratings, where behavior along the competence dimension influenced competence ratings (Table 2). In addition, warmth ratings influenced competence ratings, in that warm targets were rated as more competent. Also, the developed warmth × developed competence interaction effect was significant. The interaction revealed that warmth influenced competence ratings the most when the target was competent, so that targets that were both warm and competent were rated as most competent. Competent targets were rated as more competent on the last compared to the first trial; *M*_Diff_ = 9.92, *SD* = 12.54, *t*(71) = 6.70, *p* < .001, and incompetent targets as more incompetent on the last compared to the first trial; *M*_Diff_ = 11.83, *SD* = 12.67, *t*(71) = 7.92, *p* < .001 (Fig 6). The mean difference between the first and the last ratings were not significant between competent and incompetent targets; *t*(71) = 1.04, *p* = .301.

#### Probability ratings

**Fig 7:**
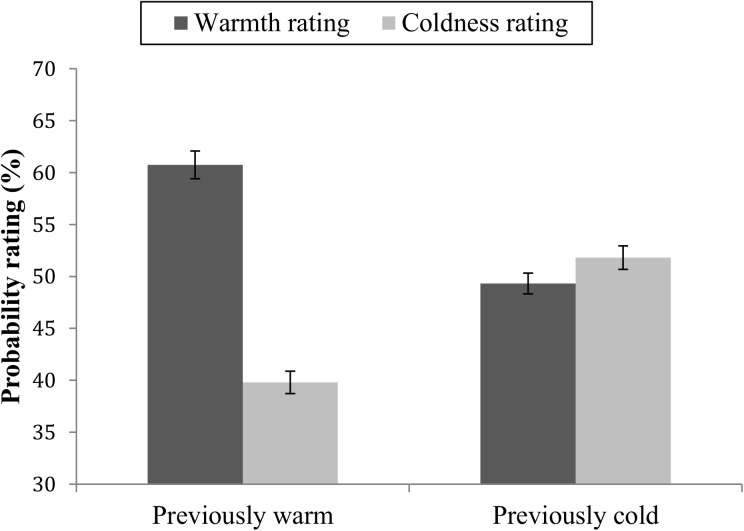
Mean probability ratings of future warm and future cold behaviors. Ratings of targets with previously mainly warm or mainly cold behavior. Error bars represent standard errors.

The design was balanced so that each participant had met four warm, four cold, four competent and four incompetent targets. But due to incomplete balancing, not all participants were exposed to all four possible warmth and competence level combinations. Forty-eight participants were exposed to all four combinations (warm and competent, warm and incompetent, cold and competent, cold and incompetent), whereas the remaining twenty-four participants were exposed to two of the four combinations (twelve participants were exposed to warm and incompetent targets and to cold and competent targets, the other twelve to warm and competent targets and to cold and incompetent targets. To probe for undesired consequence of the imbalance, we ran a missing values analysis, and tested whether the missing values were a random subset of the data. The results suggest that the data was missing completely at random (Little’s MCAR χ^2^ = .28). To account for these missing values in the dataset in analysis of both warmth and competence and possible interaction effects, a factorial linear mixed model was chosen to analyze how probability ratings were influenced by the behavioral information. For modelling purposes rating-dimension (warmth or competence), previous warmth behavior (warm vs cold) and previous competence behavior (competent vs incompetent) was treated as repeated factors, and the error covariance matrix was unstructured. Probability ratings of targets’ future behaviors were analyzed separately for the competence and warmth ratings. For the warmth ratings there was a main effect of rating valence where targets overall were rated as more likely to perform positive (warm and competent) than negative (cold and incompetent) behaviors *F*(1,74.17) = 54.25, < .001.There was no main effect of target warmth, *F*(1,65.40) = 0.17, *p* = .675, or competence; *F*(1,65.73) = 0.02, *p* = .877. The expected warmth × rating valence interaction effect was significant *F*(1,67.77) = 59.01, *p* < .001 (Fig 7). The two-way interaction effect was not qualified by a three-way warmth × competence × rating valence effect; *F*(1,71.74) = 0.94, *p* = .333. Pairwise comparisons showed that differences in probability ratings of future warm and future cold behaviors were large for targets who had previously showed warmth (*M*_diff_ = 20.92, *SE* = 2.04, *p* < .001). Differences in probability ratings were not significant among the cold targets (*M*_Diff_ = -2.49, *SE* = 1.89, *p* = .193). When studying the ratings among participants who had rated all target combinations, the absolute size of the difference in warm vs cold ratings (*M* = 19.72, *SD* = 21.26) was significantly larger for the warm than the cold targets, *t*(47) = 6.42, *p* < .001. Mirroring the results from study 1, information about warm behaviors had a strong influence on probability ratings, while information about cold behaviors had less influence on the judgments of future behavior.

In addition there was a competence × rating valence interaction effect; *F*(1,58.54) = 10.29, = .002, showing that competent targets were considered more likely to show warm than cold behaviors (*M*_Diff_ = 13.50, *SE* = 1.83, < .001) but also that incompetent targets were rated as more likely to behave warm than cold (*M*_Diff_ = 4.93, *SE* = 1.82, *p* < .001).

**Fig 8:**
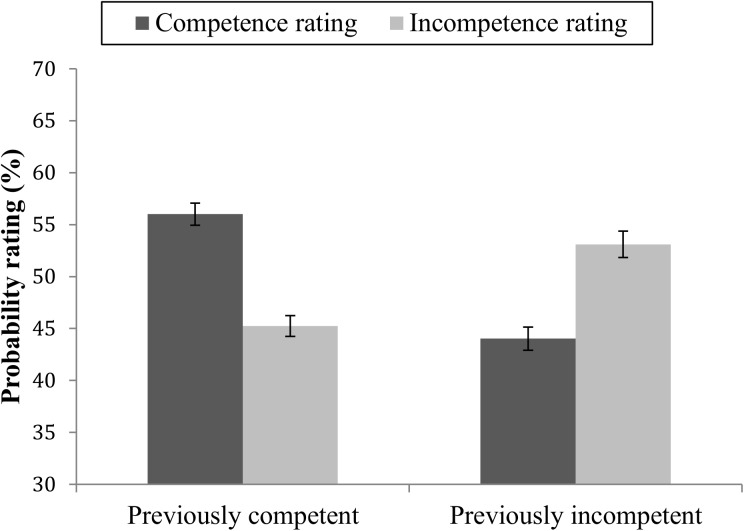
Mean probability ratings of future competent and future incompetent behaviors. Ratings of targets with previously mainly competent or mainly incompetent behavior. Error bars represent standard errors.

A linear mixed model with the same repeated factors as above was conducted for the probability ratings of future competent and incompetent behaviors. There was no main effect of rating valence; *F*(1,74.91) = 0.39, *p* = .534, and no main effect for warmth; *F*(1,59.23) = 0.13, *p* = .715. There was a main effect for competence; *F*(1,69.56) = 8.76, *p* = .004, showing that targets who had showed competence were rated somewhat more likely to perform the various behaviors (*M* = 49.72, *SE* = 0.65) compared to incompetent targets (*M* = 47.91, *SE* = 0.76). More interestingly, a competence × rating valence interaction effect was found; *F*(1,60.52) = 66.66, *p* < .001 (Fig 8). There was no significant competence × warmth × rating valence interaction effect; *F*(1,63.00) = 3.26, *p* = .075. Pairwise comparisons showed that differences in probability ratings of future competent and future incompetent behaviors were significant for targets who had previously showed competence (*M*_Diff_ = 10.76, *SE* = 1.66, *p* < .001). Differences in probability ratings were also significant among the incompetent targets (*M*_Diff_ = -9.07, SE = 1.96, *p* < .001).

A warmth × rating valence interaction effect; *F*(1,61.21) = 22.89, *p* < .001, showed that target warmth also influenced probability ratings of competent and incompetent behaviors. Targets who had shown warmth were rated as more likely to perform competent than incompetent behaviors (*M*_Diff_ = 5.57, *SE* = 1.40, *p* < .001), and cold targets were rated as more likely to perform incompetent than competent behaviors (*M*_Diff_ = -3.87, *SE* = 1.91, *p* = .046). Among participants who had rated all combinations of targets, the absolute difference in probability scores was not significant (*M* = 3.91, *SD* = 24.60, *t*(47) = 1.10, *p* = .276.

Taken together, the results from study 3 are in line with the results from study 1 and 2. More precisely, also in a context where the perceiver is presented with both warmth-related and competence-related behavioral information, the proposed asymmetry failed to appear. The failure of the classic results to appear here too, gives reason to explore further the potential boundary conditions required for the asymmetry effect to appear.

## General discussion

The present research was an attempt to reproduce some well-known impression formation effects with a new experimental paradigm, using everyday stimulus behaviors rather than extreme, presented one at a time. The results revealed no support for the negativity effect regarding cold behaviors or the positivity effect regarding competent behavior (e.g. Reeder, [10]). Cold behavior did not influence warmth ratings more than warm behaviors did, and competent behavior did not influence competence ratings more than incompetent behavior did. The asymmetry effect was lacking both in the task where participants made trait judgments based on behavioral information and in the task where they made probability ratings of future behavior based on their memory of the target person’s previous behavior. Thus, the results are not in line with the negativity models proposed by e.g. Rozin & Royzman [9] and others, and raise questions regarding which conditions must be fulfilled for the effects to appear.

One such condition may be related to the extremity of the stimulus behaviors. In the present research we deliberately avoided behaviors that diverge from what people can be expected to show in their everyday lives. The stimulus behaviors in some previous research have been quite extreme. In some research this has been intentional, such as when Skowronski and Carlston [16] manipulated extremity systematically and used “Murdered his grandmother and dumped her body in the river”. In other research it may not have been intentional. To the extent that negativity effects for warmth (and positivity effects for competence) are contingent on extreme behaviors, it may perhaps explain why there was no evidence of them in the present research. In fact, Wojciszke, Brycz and Borkenau [17] manipulated evaluative extremity for both morality related and competence related behavioral information and found that extremely evaluative behavioral information brings about a negativity effect (i.e. that immoral behaviors have particular impact) on trait ratings whereas moderately evaluative information brings about a positivity effect (i.e. that competent behaviors have particular impact). Comparing our stimuli to those of Woiciszke et al. [16], it is clear that they are moderate rather than extreme. In other words, based on these findings we would expect a positivity effect but not a negativity effect, but found neither. Instead, the most apparent finding concerned a positivity effect of warmth in the probability ratings of future behavior. The results overall do not support asymmetry effects in the opposite direction, so this result should be interpreted with caution. Even though asymmetry effects, and particularly a negativity effect, have received much attention in the impression formation literature, there are also other researchers that emphasize other aspects of the context that influence the weight perceivers put on targets’ behaviors when forming impressions. Correspondent Inference Theory (CIT; Jones & Davis, 1965 [18]) is an example of this. According to CIT, we are more inclined to make inferences regarding a target’s personality under certain conditions. For instance, when a target behaves in a way that deviates from the expected/usual behavior, this behavior is inferred to correspond more with that person’s personality compared to when a target behaves in a way that we expect most people to behave (in a situation like this). In some cases negative behaviors may be more unusual and unexpected than positive behaviors, but in other situations positive behaviors may be more unexpected than negative behaviors. In accordance with CIT, other researchers have shown that observers of a conversation find typicality of behaviors to be an important factor in how much weight they put on the information when forming an impression (Kellermann (1989[19]), and that online behavior that violates normative expectations may receive more weight D'Angelo and Van Der Heide 2016 [20]) in impression formation.

As with any study on impression formation, a number of methodological choices may have impacted the way participants formed an impression of the target persons. Regarding the first impression formation task in this study for instance, the scrollbar was kept at the rating from the last judgment the participant made of the target person. This memory aid may have influenced the judgments on the initial impression formation task, and it may also be considered a threat to external validity since we mostly do not have a recorded impression of other person stored. It is possible that this methodological choice may somehow have reduced asymmetry effects in the initial impression task. But it cannot explain the lack of asymmetry in the probability ratings of future behavior, because the raters had no memory aids in this part of the study. Future studies should explore how methodological choices (amount of behavioral information about targets, number of targets, stimulus order etc.) influence asymmetry effects in impression formation.

Another possible reason for failure to replicate results is lack of statistical power. A sensitivity analysis using G*Power3 [21] showed that using a repeated measures ANOVA with a sample size of 72 is sufficient to be able to detect small to medium effects (*f* = .13), with α = .05 and power = .80. The sample sizes in these studies therefore seem adequate to be able to detect asymmetry effects, although larger samples would further increase power.

In the present research, the contextual setting of the experimental task was different than the usual. This is a limitation in the sense that it renders direct comparison with previous research more difficult. The tradition in this research area is to have a decontextualized impression formation task. In the present research, we made an attempt to frame the task in a more specific everyday context. As there are many different possible contexts, each affording different levels of warmth and competence, the chosen context may affect the results. This was a conscious choice in the present research. We went for an every-day impression formation task, in a neighborhood setting. The neighbors were of different sex, age and ethnicity. It appeared reasonable to study how one, when recently having moved in, perceives the neighbors, and the impression that one gets of them when they show behavior with the level of warmth/coldness and that can reasonably be expected in this context. Other contexts, such as a recruitment situation, may afford other behaviors and affect the impressions that are made. The participants in these studies were all Swedish, which may be considered a limitation. Although asymmetry effects are not conceptualized in the literature as culture bound, it is possible that cultural differences also influence this kind of impression formation effects.

We used a computerized paradigm where behavioral information regarding each target person was presented sequentially, which is different from the traditional methods in the impression formation area [22]. The selection of stimulus behavior, emphasizing the behaviors that people actually perform in their lives, should increase the external validity. The fact that some well-known effects do not appear in this new paradigm is interesting. Warmth-competence research has had a revival in recent years, but it should be noted that it has focused primarily on questions related to how groups are perceived, rather than behavior-trait implications. We encourage further attempts of re-examining effects from the impression formation literature, a central area of psychology that should be given the attention that it deserves.

## Supporting information

S1 File Data study 1 ratings. Data study 1a.
(SAV)

S2 File Data study 1 probability. Data study 1b.
(SAV)

S3 File Data study 2 ratings. Data study 2a.
(SAV)

S4 File Data study 2 probability. Data study 2b.
(SAV)

S5 File Data study 3 ratings. Data study 3a.
(SAV)

S6 File Data study 3 probability. Data study 3b.
(SAV)
